# Expression of Nitric Oxide-Transporting Aquaporin-1 Is Controlled by KLF2 and Marks Non-Activated Endothelium *In Vivo*

**DOI:** 10.1371/journal.pone.0145777

**Published:** 2015-12-30

**Authors:** Ruud D. Fontijn, Oscar L. Volger, Tineke C. van der Pouw-Kraan, Anuradha Doddaballapur, Thomas Leyen, Josefien M. Baggen, Reinier A. Boon, Anton J. G. Horrevoets

**Affiliations:** 1 Department of Molecular Cell Biology and Immunology, VU Medical Center, Amsterdam, the Netherlands; 2 Institute for Cardiovascular Regeneration, Centre of Molecular Medicine, Goethe University, Frankfurt am Main, Germany; University of Amsterdam Academic Medical Center, NETHERLANDS

## Abstract

The flow-responsive transcription factor Krüppel-like factor 2 (KLF2) maintains an anti-coagulant, anti-inflammatory endothelium with sufficient nitric oxide (NO)-bioavailability. In this study, we aimed to explore, both *in vitro* and in human vascular tissue, expression of the NO-transporting transmembrane pore aquaporin-1 (AQP1) and its regulation by atheroprotective KLF2 and atherogenic inflammatory stimuli. *In silico* analysis of gene expression profiles from studies that assessed the effects of KLF2 overexpression *in vitro* and atherosclerosis *in vivo* on endothelial cells, identifies AQP1 as KLF2 downstream gene with elevated expression in the plaque-free vessel wall. Biomechanical and pharmaceutical induction of KLF2 *in vitro* is accompanied by induction of AQP1. Chromosome immunoprecipitation (CHIP) confirms binding of KLF2 to the AQP1 promoter. Inflammatory stimulation of endothelial cells leads to repression of AQP1 transcription, which is restrained by KLF2 overexpression. Immunohistochemistry reveals expression of aquaporin-1 in non-activated endothelium overlying macrophage-poor intimae, irrespective whether these intimae are characterized as being plaque-free or as containing advanced plaque. We conclude that AQP1 expression is subject to KLF2-mediated positive regulation by atheroprotective shear stress and is downregulated under inflammatory conditions both *in vitro* and *in vivo*. Thus, endothelial expression of AQP1 characterizes the atheroprotected, non-inflamed vessel wall. Our data provide support for a continuous role of KLF2 in stabilizing the vessel wall via co-temporal expression of eNOS and AQP1 both preceding and during the pathogenesis of atherosclerosis.

## Introduction

Reduced NO bioavailability is considered the hallmark of endothelial dysfunction. This pathological state of the endothelium develops in the setting of cardiovascular disease risk factors and is characterized by incomplete dilation of arteries and arterioles in response to stimuli that trigger the release from endothelial vasodilators. NO contributes to vascular homeostasis not only by stimulating vasodilation but also by negative regulation of coagulation and inflammation. Endothelial dysfunction is considered a key event in the development of atherosclerosis and predates its clinical manifestation by many years [1].

Previously, we analyzed atherosclerosis stage-specific gene expression in the endothelium from human large arteries using combined laser microbeam microdissection and microarray expression profiling. This enabled us to define endothelial signature genes for early and advanced stages of atherosclerosis. Using immunohistochemistry, increased expression of corresponding proteins in the endothelium was shown to have discriminative value for plaque-free versus early stages in case of chemokine (C-X3-C motif) ligand 1 (CX3CL1), interferon gamma-induced protein 10 (IP10/CXCL10) and T-box 18 (TBX18) and for plaque-free versus advanced stages in case of Bcl2-associated X protein (BAX), activated SMAD family member 2 (SMAD2) and nuclear factor of kappa light polypeptide gene enhancer in B-cells 2 (NFKB2). Increased expression of the latter two proteins corresponded with a significant induction of transforming growth factor beta (TGF-β)- and pro-inflammatory NF-ĸB target genes in advanced stages of atherosclerosis, as established by further *in silico* analysis of the microarray data set [2].

Notwithstanding the systemic nature of cardiovascular risk factors as dyslipoproteinemia and diabetes, atherosclerosis develops preferentially in the vessel wall at loci where endothelial cells experience severely reduced- or oscillatory shear stress. In contrast, regions of high, unidirectional laminar shear stress are relatively protected [3]. Comparative *in vitro* studies of the transcriptome of endothelial cells exposed to prolonged (≥4 days) laminar shear stress versus cells kept under static conditions identified Krüppel-like factor 2 (KLF2) as shear stress-induced transcription factor that orchestrates the anti-coagulant and anti-inflammatory transcriptome of normal, quiescent endothelium [4,5,6]. KLF2 was shown to regulate expression of slightly more than a thousand genes, most of them in an indirect manner, as could be deduced from the delayed appearance of their transcripts [5,7]. A lack of proper antibodies has thus far put constraints on detailed mapping of KLF2 expression and associated atheroprotection in human vascular tissue.

Upregulation of KLF2 transcription during prolonged exposure to shear stress requires signalling down a pathway involving mitogen-activated protein kinase kinase 5 (MEK5), extracellular-signal-regulated kinase 5 (ERK5) and myocyte enhancer binding factor 2 (MEF2) [6,8]. Pharmacological inducers of KLF2 are 3-hydroxy-3-methyl-glutaryl-coenzyme A (HMG-CoA) reductase inhibitors, better known as statins [9,10]. Mechanistically, statins inhibit geranyl-geranylation of the small GTPase Rho, and thus relieve the inhibitory effects of Rho on the ME5/ERK5/MEF2 pathway [10]. It was demonstrated that KLF2 mediates the statin-induced expression of thrombomodulin and endothelial nitric oxide synthase (eNOS), which directly contribute to an anti-inflammatory, anti-thrombotic endothelial phenotype and, in case of eNOS, vasorelaxation [11,12]. Interestingly, among KLF2 downstream genes we detected AQP1 [7], encoding a transmembrane pore protein involved in transport of water and NO [13,14] and induced by laminar shear stress in a model of wound healing [15]. These findings suggest that AQP1 might further corroborate the relation between laminar shear stress, KLF2 and a non-dysfunctional, atheroprotected endothelial phenotype by facilitating NO release.

Here, we examined the role of KLF2 in regulation of AQP1 expression and studied expression of AQP1 mRNA and protein during the pathogenesis of atherosclerosis in human vascular tissue. We provide evidence that AQP1 is a direct target gene of KLF2 and that cell-surface expressed aquaporin 1 protein marks atheroprotected endothelium *in vivo*.

## Material and Methods

### Endothelial cell culture and exposure to shear stress

Human Umbilical Vein Endothelial Cells (HUVECs) were isolated and cultured as described previously [16]. The culture medium was composed of medium 199 (Gibco, Bleiswijk, the Netherlands), supplemented with 20% (v/v) fetal bovine serum, 50 μg/ml heparin (Sigma-Aldrich), 12.5 μg/ml endothelial cell growth supplement (Sigma-Aldrich) and 100U/ml penicillin/streptomycin (Gibco). All culture surfaces were fibronectin coated.

Cells were subjected to laminar shear stress as previously described [4], with the following modifications: cells were seeded in a parallel plate type flow chamber [μ-slide I 0.6 luer (Ibidi, Martinsried, Germany) coated with fibronectin and exposed to a calibrated mean shear stress level of 18 dyne/cm^2^ during 5 days.

### Lentiviral transduction

The entire human KLF2 open reading frame was cloned behind the human phosphoglycerate kinase 1 (PGK) promoter as described previously [5]. Lentiviral short hairpin RNA constructs, targeting KLF2, were selected from the human MISSION shRNA bacterial glycerol library (Sigma-Aldrich). Vectors for KLF2 overexpression and KLF2 silencing were packaged into lentiviral particles in HEK293T cells as described [16] using the CalPhos mammalian transfection kit (Clontech, Palo Alto, CA). HUVECs in first or second passage were trypsinized, replated at 80% confluence and transduced overnight in complete culture medium.

### Real-time Reverse Transcription-Polymerase Chain Reaction [RT-PCR]

Total RNA was isolated using the Aurum total RNA mini kit (Bio-Rad, Veenendaal, The Netherlands) according to the manufacturer’s instructions. 0.5 μg of total RNA was used for reverse transcription, with an (dT)12-18 primer using a RevertAid H-minus first strand cDNA synthesis kit (Thermo Scientific, Bleiswijk, the Netherlands), according to the manufacturer’s instructions. PCR reactions were performed in 10 μl volumes using Fast SYBR Green Master Mix (Life Technologies, Bleiswijk, the Netherlands) on an StepOne real-time PCR system (Life Technologies) as described [17]. Specificity of the amplification was checked by melt curve analysis. P0 levels were used for normalization. Primer sequences: AQP1, 5’-GGATTAACCCTGCTCGGTCC-3’ and 5’-CCCACCCAGAAAATCCAGTG-3’, P0, 5’-TCGACAATGGCAGCATCTAC-3’ and 5’-ATCCGTCTCCACAGACACAAGG-3’, eNOS, 5’-GCGGCTGCATGACATTGAG-3’, and 5’-TCGTCGCGGTAGAGATGGTC-3’.

### Overexpression of KLF2V5 and chromatin immunoprecipitation (ChIP)

HUVECs were transduced with lentivirus encoding V5-tagged KLF2 or control virus and immunoprecipitation with V5 antibody (ab9116, Abcam, Cambridge UK) was carried out essentially as described [18]. To generate the lentivector, KLF2 cDNA without stop-codon was cloned in pENTR-D-TOPO (Life Technologies) and subcloned in pLenti4-V5-Dest (Life Technologies). The precipitated chromatin fragments were subjected to RT-PCR, using primers specific for regions that contain putative KLF-binding sites. Primer sequences: 378–464 bp upstream of AQP1 startcodon, 5’-CAACGTCCACCCACTGAGTC-3’, 5’-GTGCAAAGCTCAGGGAGTTC-3, 33–152 upstream AQP1 startcodon, 5’-CCAGAGGAGGTCTGTGTGGT-3’, 5’-GGGTGCTCAATTCCCTCTG-3’.

### Collection of human vasculature and classification of atherosclerosis

Human large arteries were collected post-mortem after disease, in compliance with institutional guidelines, as described in a previous study [2]. Written informed consent for the use of tissue in research was obtained and the use of tissue and patient data was in agreement with the Dutch “Code of Conduct for Responsible Use” (2011) (https://www.federa.org). The atherosclerotic plaques were classified according to Virmani, [19]. The main criterion for discriminating between early and advanced plaques was the presence of a fibrous cap. Integrity of the endothelium was checked by immunohistochemistry for CD31 (clone JC70A, Dako, Glostrup, Denmark).

### Immunohistochemistry

Individual cross-sections were incubated with antibodies against aquaporin-1 (clone 1/A5F6, Abcam, Cambridge, UK), ICAM-1 (clone My13, Zymed, South San Francisco, CA), CD31 (clone JC70A, Dako, Glostrup, Denmark), monocyte/macrophage (clone HAM56, Dako), actin (smooth muscle)(clone 1A4, Dako). All antibodies were diluted in TBS containing 1% weight/volume (w/v) bovine serum albumin (BSA), (Sigma-Aldrich, Zwijndrecht, the Netherlands) and 0.01% (w/v) Tween-20 (Sigma-Aldrich). The incubations with secondary biotin-conjugated antibodies (Dako) were followed by amplification with a streptavidin-HRP complex (Dako), and a peroxidase-substrate staining (Nova Red kit, Vector Labs, Burlingame, CA).

### Statistical analysis

Data are reported as mean and standard error of the mean(SEM). Differences in mean values were analyzed using Student’s t-test. Differences were considered significant at the P<0.05 level.

## Results

### AQP1 is preferentially expressed in endothelium overlying plaque-free intimae *in vivo*, and is induced by KLF2 *in vitro*

**Fig 1:**
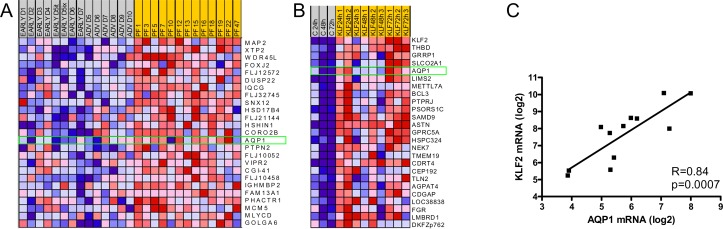
AQP1 is preferentially expressed in endothelium overlying plaque-free intimae and is induced by KLF2. (A, B) Heatmaps of the top 25 ranking genes as determined by GSEA. (A) Gene expression in endothelium overlying plaque-free (PF) intimae from human large arteries is compared to gene expression in endothelium from early- and advanced (ADV) lesions. Data set from Volger et al. [2]. Aquaporin 1 (AQP1) is identified as the highest ranking membrane-expressed gene in plaque-free lesions (green rectangle). (B) Time courses (24, 48 and 72 hours) of gene expression in mock-transduced HUVEC (control, c) and KLF2-transduced HUVEC are compared. Data set from Boon et al. [7]. The position of AQP1 is indicated by a green rectangle. (C) Correlation of KLF2—and AQP1 transcript levels during time courses (24, 48 and 72 hours) of gene expression in mock- and KLF2- transduced HUVECs (same data set as used for Fig 1B).

In a dataset comprising transcriptome data from endothelium overlying normal intimae and intimae containing early- and advanced lesions in human large arteries [2], we determined the top 25 ranking genes in a comparison between the plaque-free situation and early- and advanced lesions using Gene Set Enrichment Analysis. AQP1 was identified as the highest ranking endothelial cell-surface exposed signature gene in the plaque-free situation (Fig 1A). Likewise, we identified AQP1 as the fourth ranking gene in a comparison between transcriptomes of endothelial cells that were lentivirally transduced with either a mock- or KLF2 encoding construct and subsequently cultured for different periods of time [7] (Fig 1B).A further analysis of transcript levels during this time course revealed a significant (p = 0.0007) positive correlation between KLF2- and AQP1 mRNA (Fig 1C), suggesting that AQP1 might be part of the repertoire of KLF2-downstream atheroprotective genes. This idea is consistent with the observed preferential expression of AQP1 in endothelium overlying healthy intimae. Together, these *in silico* analyses qualify AQP1 as a potential cell-surface marker for healthy, non-dysfunctional endothelium.

### Induction of AQP1 mRNA by shear stress and statins is KLF2 dependent

**Fig 2:**
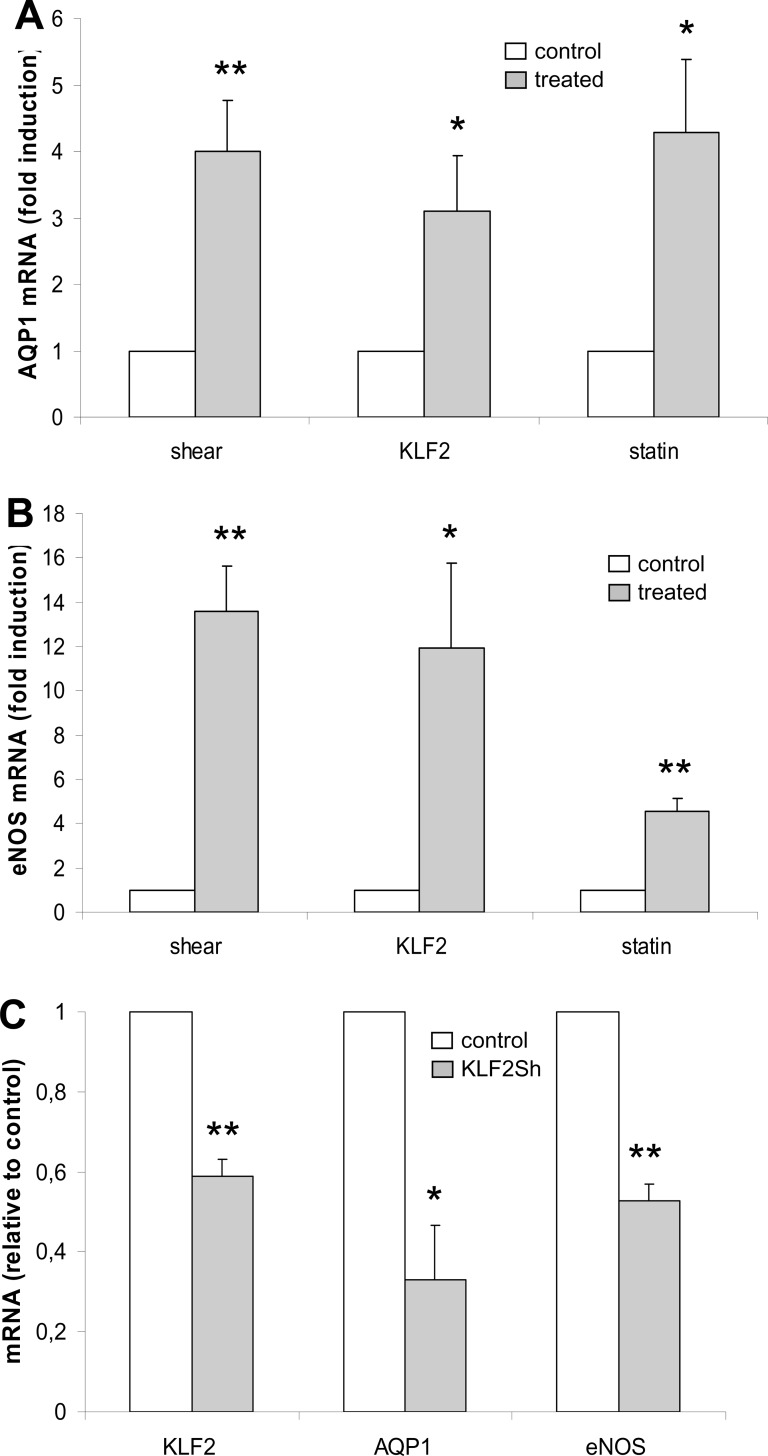
KLF2 dependency of AQP1 expression. (A, B) Expression of KLF2 was induced in HUVEC by laminar flow (shear), lentiviral transduction (KLF2) or incubation with atorvastatin (statin). mRNA levels were determined by semi-quantitative RT-PCR for AQP1 (A) and eNOS (B), normalized to P0 and shown as mean and SEM of the fold induction (grey) relative to the corresponding control (white). The following conditions are depicted: shear; exposure to laminar shear stress for ≥ 4 days at an average of 18 dyne/cm^2^, induction relative to static control (N = 6), KLF2; transduction with a lentiviral vector carrying KLF2 under control of the PGK promoter and subsequent growth for ≥ 4 days, induction relative to mock transduced cells (N = 7), statin; incubation with atorvastatin at a final concentration of 10 μM during 24 hours, induction relative to vehicle control (N = 5). *P<0.05, **P<0.01. (C) HUVECs were transduced with a lentiviral vector encoding an shRNA targeting KLF2. 24 hours later, expression of KLF2 was induced by incubation with atorvastatin at a final concentration of 10 μM during 24 hours. mRNA levels were determined for KLF2, AQP1 and eNOS, normalized to P0 and shown as mean and SEM mRNA level (grey) relative to control cells transduced with a non-targeting construct (white)(N = 3). *P<0.05, **P<0.01.

We studied the effect of KLF2 expression on AQP1 mRNA levels. Either KLF2 was constitutively overexpressed from a lentiviral vector, or KLF2 was induced by mechanical or pharmaceutical stimuli. The following conditions were applied: 1. Cells were exposed to prolonged laminar shear stress (≥ 4 days at an average of 18 dyne/cm^2^) [4]. 2. Cells were transduced with a lentiviral vector expressing KLF2 from the phosphoglycerate kinase-1 promoter and subsequently grown for ≥ 4 days [5]. 3. Cells were incubated with atorvastatin at a final concentration of 10 μM during 24 hours [9,10]. Total RNA was harvested and used to determine induction of AQP1 by means of semi-quantitative RT-PCR. As a control, we determined induction levels of an established KLF2 direct target gene; eNOS. Compared to the other conditions applied, duration of statin incubation was limited to 24 hours due to adverse effects of prolonged cholesterol-deprived growth that results from HMG-CoA inhibition. Despite differences in duration of stimulation, AQP1 showed a significant upregulation of between three- and four-fold with no significant differences between applied stimuli (Fig 2A). eNOS showed a comparable, significant five-fold upregulation during 24 hours culture in the presence of statins but tended to be higher upregulated under conditions of prolonged shear (fourteen-fold) or ≥4 days of KLF2 overexpression (twelve-fold), which is in full agreement with earlier studies of our group [4,5] (Fig 2B). These results reveal that AQP1 mRNA is induced within 24 hours of KLF2 induction by statin, suggesting a direct role of KLF2 in AQP1 transcription. Furthermore, the data suggest that AQP1 mRNA steady state levels are reached within a shorter time-span as compared to eNOS mRNA.

Dependence of AQP1 expression on KLF2 was further assessed by KLF2 silencing under conditions of pharmacological KLF2 induction. AQP1 and eNOS transcription were repressed upon KLF2 silencing, indicating that AQP1 as well as eNOS is subject to transcriptional regulation by KLF2 (Fig 2C).

Taken together, these results show that induction of KLF2 by mechanical and pharmacological means is accompanied by upregulation of AQP1 mRNA expression and that KLF2 affects AQP1 transcription via a direct positive regulatory mechanism.

### KLF2 directly binds the AQP1 promoter

**Fig 3:**
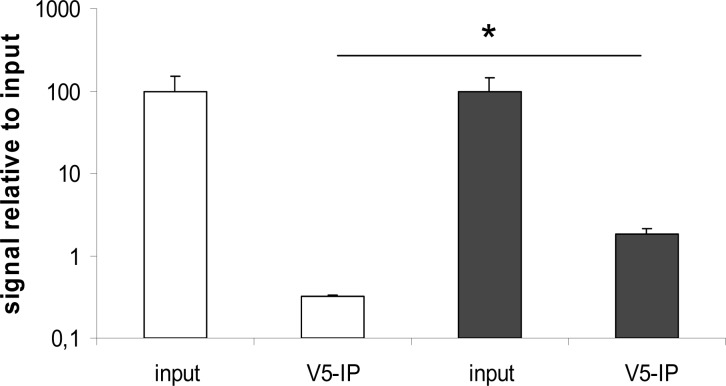
KLF2 binding to the AQP1 promoter. HUVECs were transduced with control virus (white) or lentivirus encoding V5-tagged KLF2 (black). Immunoprecipitation was performed using an anti-V5 tag antibody. RT-PCR was carried out on the precipitated chromatin using primers specific for the regions containing putative KLF2-binding sites on the AQP1 promoter (N = 3). *P<0.05.

To investigate whether KLF2 interacts with the AQP1 promoter, we analyzed the AQP1 promoter *in silico* for putative KLF binding sites, using rVISTA (S1 Fig). Chromatin immunoprecipitation and subsequent semi-quantitative RT-PCR revealed a 6-fold enrichment of the KLF-binding site between position -422 and -435 (relative to the AQP1 startcodon), in KLF2-V5 transduced cells relative to control-transduced cells (Fig 3). These results provide support for direct transcriptional activation of the AQP1 promoter by KLF2 and identify AQP1 as a direct target gene of KLF2.

### Suppression of AQP1 transcription by inflammatory TNF-α is restrained by KLF2

**Fig 4:**
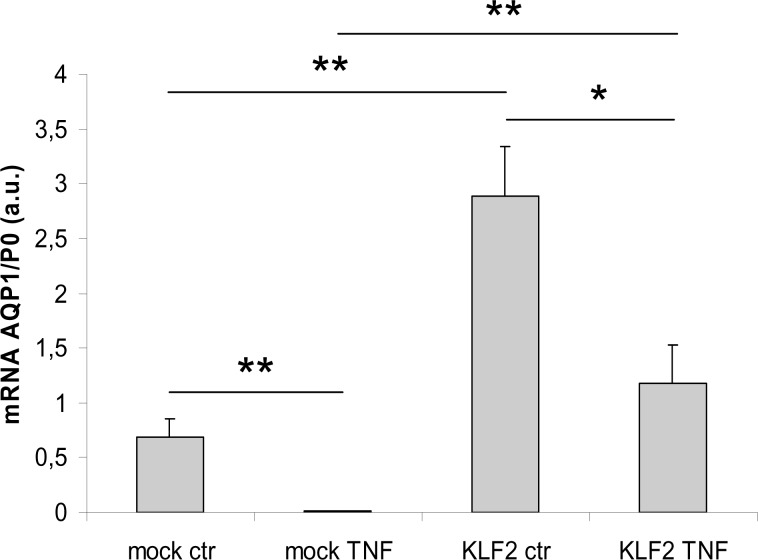
Suppression of AQP1 transcription by TNF-α is restrained by KLF2. HUVECs were transfected with either empty lentiviral vector (mock) or with a lentiviral vector carrying KLF2 under control of the PGK promoter (KLF2) and two days after transfection incubated with vehicle (ctr) or TNF-α at a concentration of 20 ng/ml during 24 hours (TNF). mRNA levels were determined for AQP1, normalized to P0 and expressed as mean and SEM (N = 4). *P<0.05, **P<0.01.

In contrast to the atheroprotective effects of shear stress and KLF2 expression, intimal inflammation is a driving force in the pathology of atherosclerosis. We examined the response of AQP1 transcription to the inflammatory mediator TNF-α. HUVECs, either mock-transduced or transduced with an KLF2 encoding lentiviral vector, were incubated with 20 ng/ml TNF-α during 24h. As expected, levels of overexpressed KLF2 mRNA did not significantly change upon TNF-α incubation (S2A Fig). Basal expression levels of both AQP1 and eNOS were significantly reduced under TNF-α-induced inflammatory conditions. Constitutive overexpression of KLF2 completely preserved eNOS mRNA expression upon TNF-α incubation, (S2B Fig), whereas AQP1 mRNA expression was partially maintained (Fig 4). These results indicate that AQP1 transcription is reduced under TNF-α mediated inflammatory conditions and that KLF2 is able to significantly counteract this reduction.

### Endothelial AQP1 expression is lost specifically in endothelium overlying inflamed atherosclerotic plaques

*In silico* analysis of gene expression profiles from studies that assessed the effects of, respectively, KLF2 overexpression *in vitro* and atherosclerosis *in vivo* on endothelial cells, identified AQP1 as KLF2 downstream gene with elevated expression in the plaque-free vessel wall. (Fig 1A). Consistently, our *in vitro* data showed that AQP1 expression is subject to KLF2-mediated positive regulation under atheroprotective conditions (statins, shear stress) and is downregulated under atherogenic inflammatory conditions (Fig 2A and 2C, Fig 4).

Using immunohistochemistry, we subsequently verified whether the atherosclerosis stage-related differences in AQP1 mRNA levels correspond with differential expression of the AQP1 protein. In addition, we examined a possible effect of local inflammatory conditions on AQP1 expression, by staining sections for macrophages using HAM56. In case of positive HAM56 stain, sequential sections were stained for intercellular adhesion molecule 1 (ICAM-1). Endothelial ICAM-1 expression is known to be elevated at inflammatory cell infiltration sites of atherosclerotic plaques [20], and ICAM-1 has been implicated in promoting atherogenesis in mouse models of atherosclerosis [21,22,23]. Accordingly, in human lesions, ICAM-1 was identified as one of the signature genes for early stage plaques in our transcriptome analyses [2]. Immunohistochemistry was performed on Virmani-classified human large artery specimen that essentially comprised three subsets, characterized as either having no histological signs of atherosclerosis, or having focal atherosclerosis of the early- or the advanced stage, respectively. The focal nature of the plaques allowed paired comparison, within a single section, of endothelium overlying intimal areas either with- or without plaque. As check for endothelial integrity, sections were stained with CD31 (data not shown).

#### Plaque free vascular specimen

**Fig 5:**
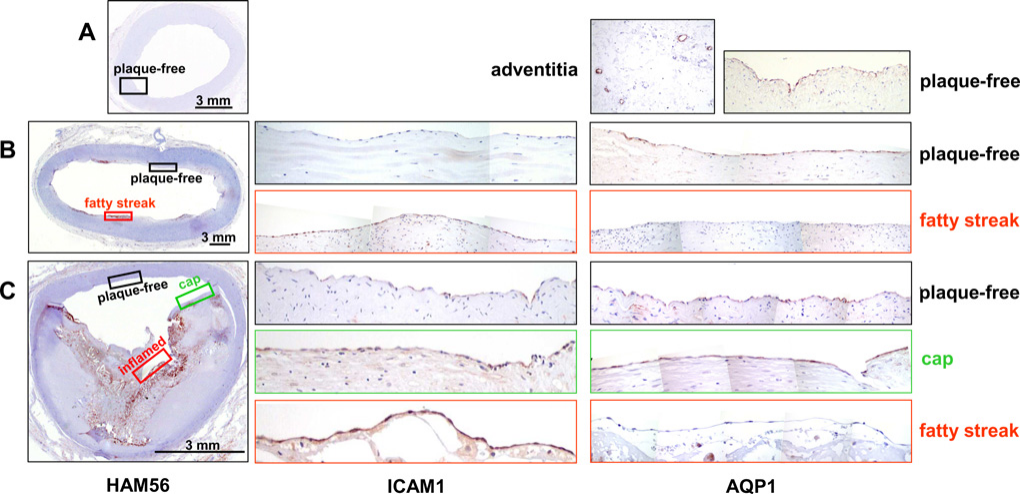
AQP1 immunohistochemistry in vascular tissue specimen showing different stages of atherosclerosis. Overviews, showing immunohistochemistry for macrophages (HAM56, left column), are given for arteries without lesions (A, external iliac artery), with focal lesions of the initial stage (B, abdominal aorta, intimal xanthoma/fatty streak) or the advanced stage (C, common iliac artery, fibro-calcific plaque with signs of rupture). Rectangles within the HAM56 overview indicate the position of areas that are shown as magnification of serial sections stained for ICAM-1 (middle column) and AQP1 (right column). In addition, these sections/areas were further characterized with regard to their cellular composition by staining for macrophages (HAM56) and smooth muscle cells (anti α-actin) (S3–S5 Figs).

Fig 5A shows a section of an external iliac artery without histological signs of atherosclerosis and negative for macrophage staining with HAM56 antibody. A homogenous AQP-1 immunostain was observed on endothelial cells lining the lumen. This observation is in agreement with microarray mRNA data for AQP1 that identify AQP1 as signature gene for the plaque-free situation (Fig 1A). Furthermore, AQP1 expression was abundant on endothelial cells lining capillaries within adventitial layers (Fig 5A).

#### Specimen containing early lesions

Fig 5B shows a section of an abdominal aorta containing focal early lesions (intimal xanthoma/ fatty streak). Intimal HAM56/macrophage stain was specifically associated with these lesions and the overlying endothelium showed an elevated ICAM-1 stain as compared to the endothelium from plaque-free regions (S3 Fig and Fig 5B).This finding corresponds well with our microarray data that identify the ICAM-1 transcript as marker for early lesions [2]. AQP1 immunostain was detected in lesion-free areas and was lost from the activated, ICAM-1 positive endothelium overlying the macrophage-rich early lesion.

#### Specimen containing advanced lesions

As shown in Fig 5C, the vessel wall of this common iliac artery comprises plaque-free areas as well as areas with characteristics of advanced lesions (fibro-calcific plaque with signs of rupture). Interestingly, within advanced atherosclerotic lesions the endothelial stain of ICAM-1 and AQP1 showed a heterogenic distribution and seemed to vary, comparable to the situation in early lesions, with the local inflammatory burden. Indeed, further characterization of advanced lesions revealed two basically different lesion types, i.e. macrophage-rich/SMC-poor and macrophage-poor/SMC-rich, further designated “inflamed” and “cap”, respectively (Fig 5C, S4 and S5 Figs). Subsequent analysis of endothelial ICAM-1 and AQP1 expression in plaque-free areas and inflamed- or capped advanced lesions revealed abundant expression of ICAM-1 in the advanced, inflamed lesion. In contrast, AQP1 was completely absent from advanced, inflamed lesions whereas non-inflamed areas, either characterized as advanced plaque or as being plaque-free, show positive AQP1 stain (Fig 5C). These observations reveal that AQP1 expression is not limited to the plaque free situation but is extended to macrophage-poor advanced plaques and thus further specify aquaporin-1 expression as being associated with non-activated endothelium.

## Discussion

Here, we characterize AQP1, encoding an NO-transporting transmembrane pore, as a direct KLF2 downstream gene that is specifically expressed in non-activated endothelium overlying normal- and macrophage-poor atherosclerotic intimae. Thus, AQP1 expression marks the atheroprotected, non-inflamed vessel wall.

Aquaporin-1 is an intrinsic membrane protein able to form homotetrameric water-permeable membrane pores. Its expression has been reported for capillary endothelial cells as well as for aortic endothelium [24,25,26], which is confirmed by our current data. Functionally, endothelial aquaporin-1 has mainly been described as a passive, osmolarity-driven water pore. Studies in AQP-1 null mice revealed impaired tumor angiogenesis and endothelial cell migration, which was ascribed to a decreased water influx that impairs the formation of lamellipodia at the leading edge of migrating cells [27,28,29,30]. Aquaporin-1 has recently been identified as a transporter of NO across cell membranes [13] and impairment of NO-dependent vasorelaxation, associated with decreased endothelial NO efflux, has been observed in aortic rings of AQP1 -/- mice [14]. In contrast, a recent study of Montiel et al. using both systemic and *in vitro* approaches to study vasorelaxation in AQP1 -/- mouse reports intact NO-dependent vasorelaxation [25]. So, although the ability of AQP-1 to transport NO across cell membranes seems convincingly established *in vitro*, its physiological significance is still debated. Based on findings of Herrera et al [14,15], reduced AQP1 expression in endothelium associated with inflamed intimae might result in reduced local NO availability and, consequently, impaired vasorelaxation and reduced negative regulation of coagulation and inflammation. Interestingly, both AQP1 and eNOS are direct target genes of KLF2, suggesting functional relevance of the observed co-temporal transcriptional control in endothelial NO secretion.

For immunohistochemistry, we used a set of human large artery tissue specimen originating from different types of arteries. This diversity limits possible confounding effects of endothelial heterogeneity [31] on basal expression of aquaporin-1 and its responsiveness to local atherogenic and atheroprotective stimuli. In addition, paired comparison, within a single section, of both ICAM-1 and aquaporin-1 expression in endothelium overlying normal- versus atherosclerotic intimae, further excluded confounding factors and enabled us to specifically relate local loss of aquaporin-1 expression to endothelial inflammatory activation associated with successive stages of atherosclerosis. In both early- and advanced lesions, macrophage-rich areas were overlayed by ICAM-1 positive endothelium. Concomitantly, aquaporin-1 expression was absent from the activated endothelium. In contrast, areas showing no histological signs of atherosclerosis as well as advanced, macrophage-poor lesions invariably showed consistent aquaporin-1 expression in endothelial cells. These observations reveal specificity of aquaporin-1 expression for non-activated endothelium and confirm, at the protein level, the atherosclerosis stage-related differences in AQP1 mRNA expression found in our *in silico* analysis. In addition, immunohistochemistry shows that expression of aquaporin-1 is not strictly confined to endothelium overlying plaque-free areas but also includes relatively small areas of the vessel wall that show lesions from which macrophages are absent.

Our *in vitro* experiments show that AQP1 transcription is under direct positive control of the atheroprotective transcription factor KLF2 and, in response to KLF2, is upregulated upon statin treatment and under laminar shear stress. In contrast, AQP1 transcription is repressed in the presence of the inflammatory mediator TNF-α and constitutive overexpression of KLF2 is able to restrain this repression even at high concentrations of TNF-α. In good agreement, immunohistochemistry shows, at the protein level, that *in vivo* expression of AQP1 is intimately associated with non-activated, atheroprotected, endothelium whereas expression is absent in activated, ICAM-1 positive endothelium overlying macrophage-rich intimae.

Detailed mapping of KLF2 expression and associated atheroprotection in human vascular tissue still awaits the availability of proper KLF2 antibodies. Yet, aquaporin-1 signals observed in endothelium overlying macrophage-poor plaques suggest that expression of KLF2 occurs even in advanced stages of atherosclerosis, provided that the balance between local inflammatory burden and local shear stress is in favour of the latter. Therefore, the possibility should be considered that KLF2 exerts atheroprotective effects in the endothelium beyond the onset of atherosclerosis and may thus contribute to plaque stabilization.

Collectively, our data show that laminar shear-stress and statins provide luminal cues that induce AQP1 expression in an KLF2-dependent manner. Inflammatory processes at the intimal side of the endothelium critically repress AQP1. Thus, AQP1 expression characterizes atheroprotected endothelium overlying non-inflamed intimae in the atherosclerotic- as well as in the normal vessel wall.

## Supporting Information

S1 Fig KLF is predicted to bind the AQP1 promoter region. Illustration and sequence of the genomic region 515 bp upstream- and 486 bp downstream of the AQP1 start codon (green). Putative KLF binding sites indicated in red.
(PDF)

S2 Fig Effect of inflammatory TNF-α on KLF2 overexpression and induced eNOS expression. HUVECs were transfected with either empty lentiviral vector (mock) or with a lentiviral vector carrying KLF2 under control of the PGK promoter (KLF2) and two days after transfection incubated with vehicle (ctr) or TNF-α at a concentration of 20 ng/ml (TNF) during 24 hours. mRNA levels were determined for KLF2 and eNOS, normalized to P0 and expressed as mean and SEM (N = 4). *P<0.05, **P<0.01.
(PDF)

S3 Fig Immunohistochemical characterization of vascular tissue specimen showing different stages of atherosclerosis: macrophages, HAM56. An overview, showing immunohistochemistry for macrophages (HAM56),is given for an artery with focal lesions of the initial stage (abdominal aorta, intimal xanthoma/fatty streak). Rectangles within the HAM56 overview indicate the position of areas that are shown as magnification of serial sections stained for macrophages (HAM56).
(PDF)

S4 Fig Immunohistochemical characterization of vascular tissue specimen showing different stages of atherosclerosis: smooth muscle cell, SM α-actin. An overview, showing immunohistochemistry for macrophages (HAM56), is given for an artery with focal lesions of the advanced stage (common iliac artery, fibro-calcific plaque with signs of rupture). Rectangles within the HAM56 overview indicate the position of areas that are shown as magnification of serial sections stained for smooth muscle cells (anti α-actin).
(PDF)

S5 Fig Immunohistochemical characterization of vascular tissue specimen showing different stages of atherosclerosis: macrophages, HAM56. An overview, showing immunohistochemistry for macrophages (HAM56), is given for an artery with focal lesions of the advanced stage (common iliac artery, fibro-calcific plaque with signs of rupture). Rectangles within the HAM56 overview indicate the position of areas that are shown as magnification of serial sections stained for macrophages (HAM56).
(PDF)
