# Supplementary material for: Expression of Nitric Oxide-Transporting Aquaporin-1 Is Controlled by KLF2 and Marks Non-Activated Endothelium In Vivo
Source: PLoS One. 2015 Dec 30;10(12):e0145777. doi: 10.1371/journal.pone.0145777 (PMC4696733; doi:10.1371/journal.pone.0145777)
Supplement: S1 Fig — Illustration and sequence of the genomic region 515 bp upstream- and 486 bp downstream of the AQP1 start codon (green). Putative KLF binding sites indicated in red. (PDF) [file pone.0145777.s001.pdf]

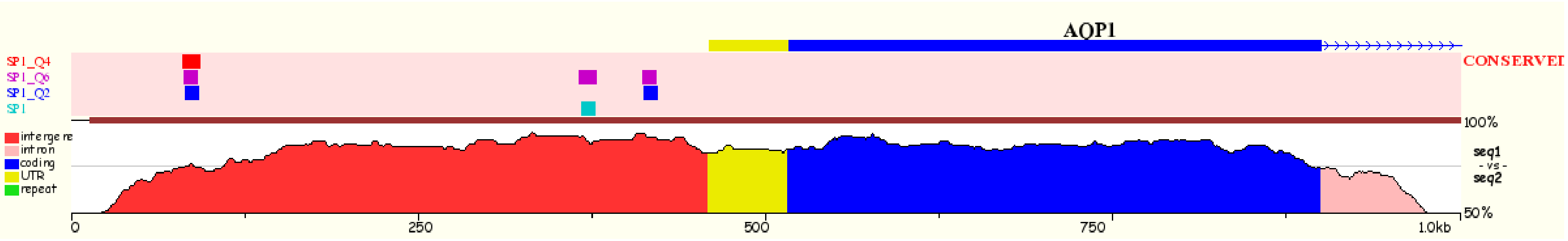

> hg18 chr7:30917535-30918538 1004bps GC:62.9%
GGGATGGGGATGTCAGGCCAGCAGGATCATGGTACCTGCCTCCAGGGAGGGCAACGTCCACCCACTGAGTCCAGCCCGCCCACCCCACCCACTCCGGAGCACCTGGCTCTG
CCCTCAGGAACTCCCTGAGCTTTGCACACAGGGCCGAGACACCTGGATTTCTCTGGTTCCCTGAGTGGGGCCAGCTTGGAAGAATTTCCCAAAGCCTATTAGAGCAACGGCT
GCCTCCTGCCTGCCTCCTTGGGCTGGGCAGGGCTGAGGGCGGAGGGAGAGAGAGAGAGAGGGAGGGGGAGAGGAGGAAGGAAAAAGTTGGCAGGCCGACAGCACAGC
CGTGTCTGCATCCATCCAGAGGAGGTCTGTGTGGTGTGGGGCGGGCCAGGAGCGAAGAGAGGCCTTCCTCCCTTTGTGCTCCCCCCGCCCCCCGGCCCTATAAATAGGCCC
AGCCCAGGCTGTGGCTCAGCTCTCAGAGGGAATTGAGCACCCGGCAGCGGTCTCAGGCCAAGCCCCCTGCCAGCATGGCCAGCGAGTTCAAGAAGAAGCTCTTCTGGAGG
GCAGTGGTGGCCGAGTTCCTGGCCACGACCCTCTTTGTCTTCATCAGCATCGGTTCTGCCCTGGGCTTCAAATACCCGGTGGGGAACAACCAGACGGCGGTCCAGGACAAC
GTGAAGGTGTCGCTGGCCTTCGGGCTGAGCATCGCCACGCTGGCGCAGAGTGTGGGCCACATCAGCGGCGCCCACCTCAACCCGGCTGTCACACTGGGGCTGCTGCTCAG
CTGCCAGATCAGCATCTTCCGTGCCCTCATGTACATCATCGCCCAGTGCGTGGGGGCCATCGTCGCCACCGCCATCCTCTCAGGCATCACCTCCTCCCTGACTGGGAACTCGC
TTGGCCGCAATGACGTGAGTGGGGTGTCCCTGGGCTTGGGGGGGTTCTAGAATGATGCTGAAAGGCACTGGTTCCATCCTCTGCCCATTGTGCAGATGGGGACACTGAGG
AACGGAGAG

S1 Fig.
