# Supplementary figures and images for: Expression of Nitric Oxide-Transporting Aquaporin-1 Is Controlled by KLF2 and Marks Non-Activated Endothelium In Vivo
Source: PLoS One. 2015 Dec 30;10(12):e0145777. doi: 10.1371/journal.pone.0145777 (PMC4696733; doi:10.1371/journal.pone.0145777)

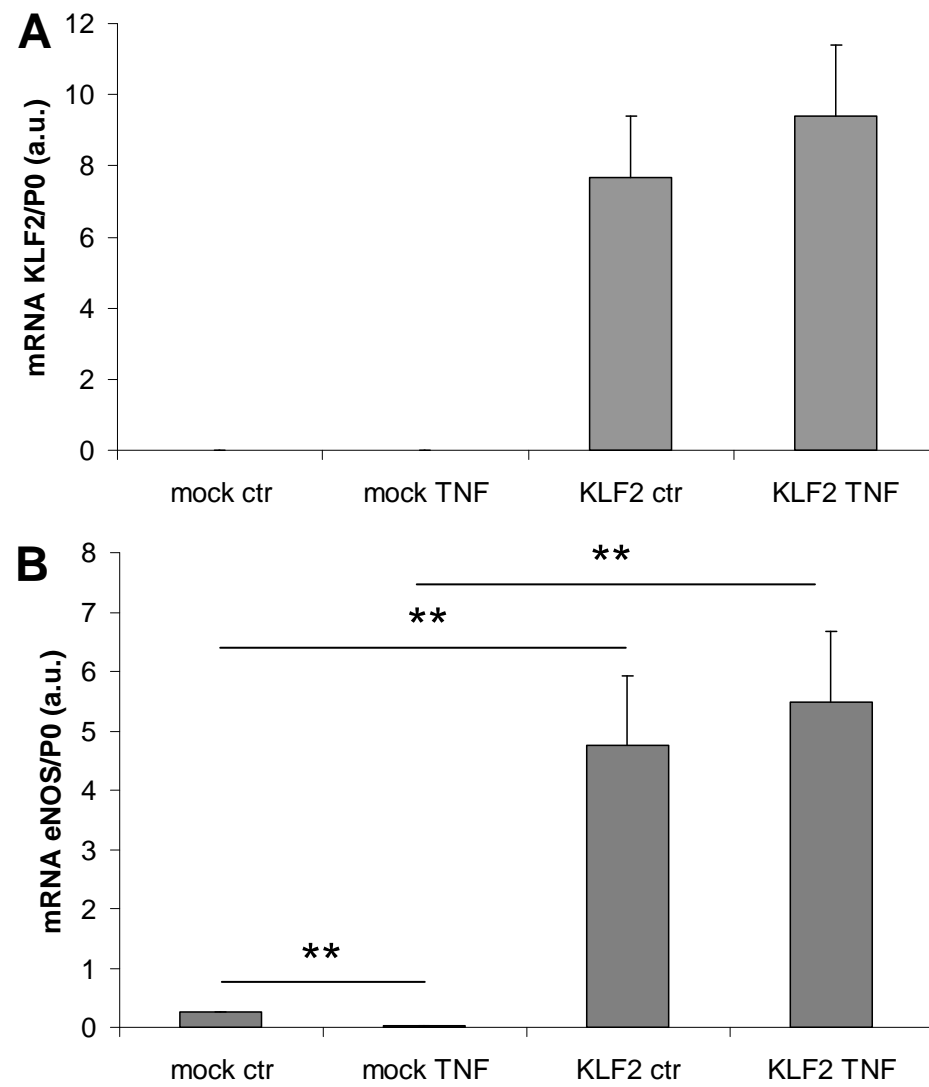

S2 Fig.

Supplement: S2 Fig — HUVECs were transfected with either empty lentiviral vector (mock) or with a lentiviral vector carrying KLF2 under control of the PGK promoter (KLF2) and two days after transfection incubated with vehicle (ctr) or TNF-α at a concentration of 20 ng/ml (TNF) during 24 hours. mRNA levels were determined for KLF2 and eNOS, normalized to P0 and expressed as mean and SEM (N = 4). *P<0.05, **P<0.01. (PDF) [file pone.0145777.s002.pdf]

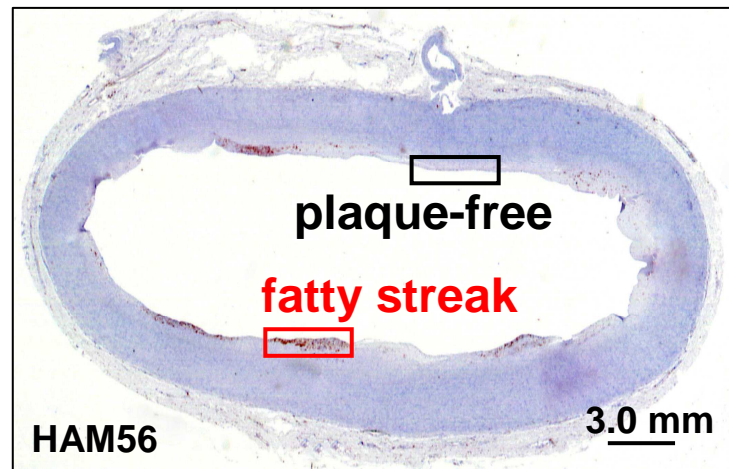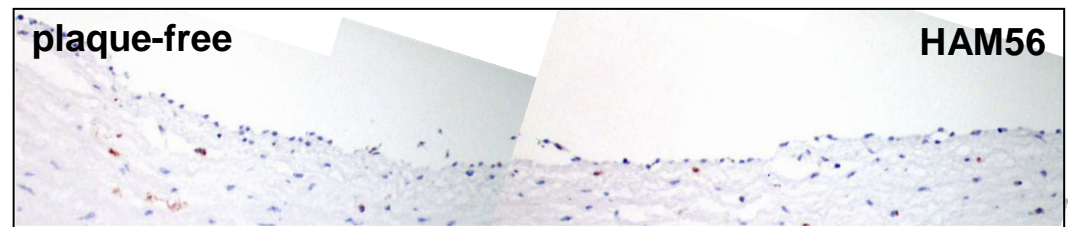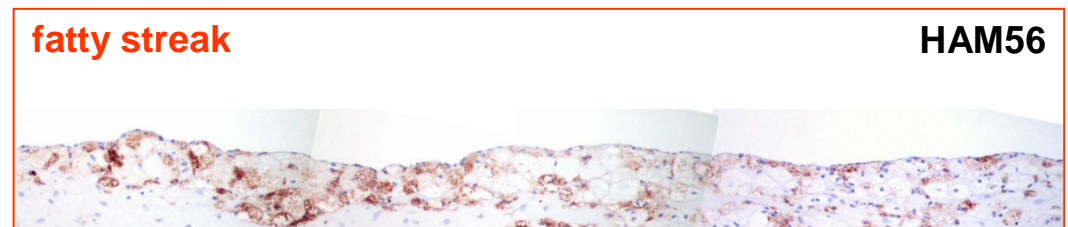

S3 Fig.

Supplement: S3 Fig — An overview, showing immunohistochemistry for macrophages (HAM56),is given for an artery with focal lesions of the initial stage (abdominal aorta, intimal xanthoma/fatty streak). Rectangles within the HAM56 overview indicate the position of areas that are shown as magnification of serial sections stained for macrophages (HAM56). (PDF) [file pone.0145777.s003.pdf]

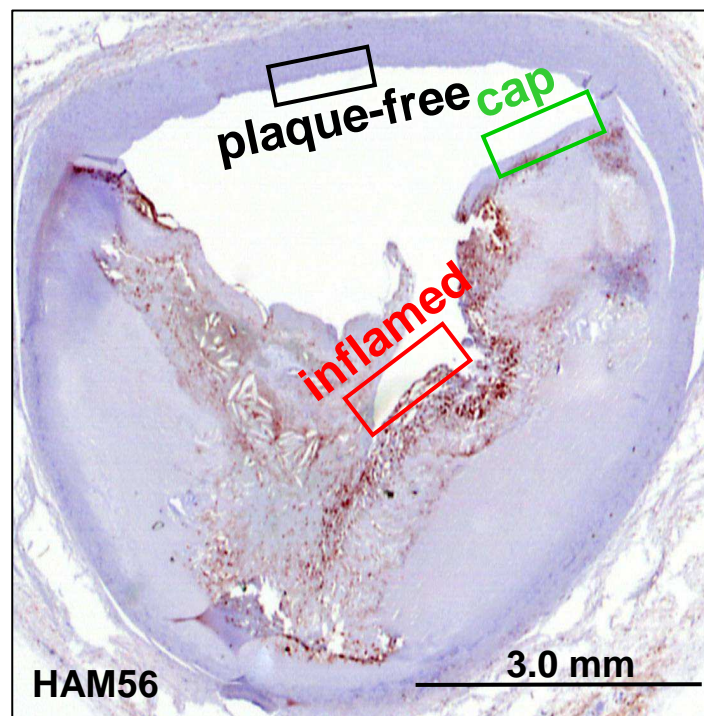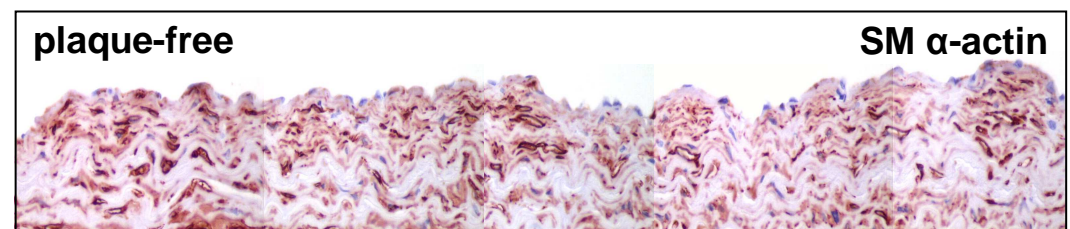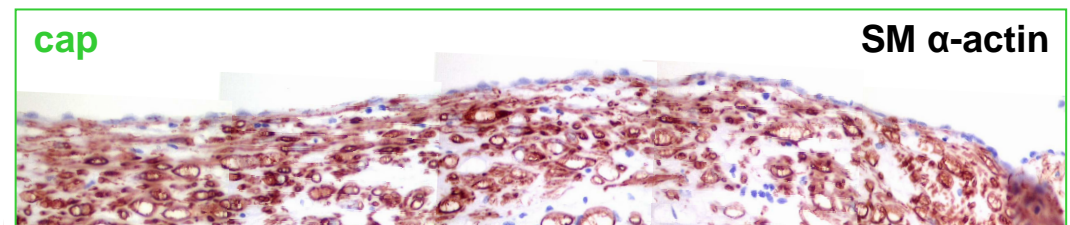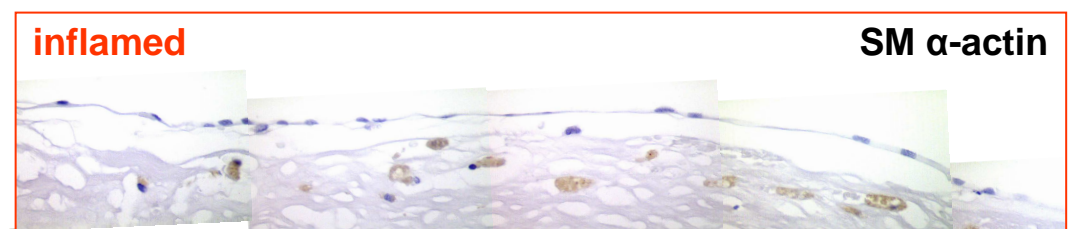

S4 Fig.

Supplement: S4 Fig — An overview, showing immunohistochemistry for macrophages (HAM56), is given for an artery with focal lesions of the advanced stage (common iliac artery, fibro-calcific plaque with signs of rupture). Rectangles within the HAM56 overview indicate the position of areas that are shown as magnification of serial sections stained for smooth muscle cells (anti α-actin). (PDF) [file pone.0145777.s004.pdf]

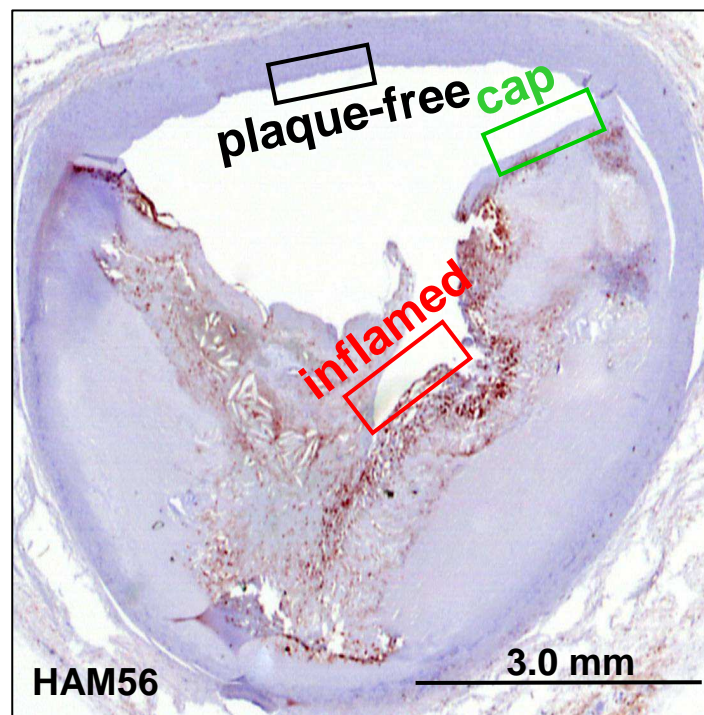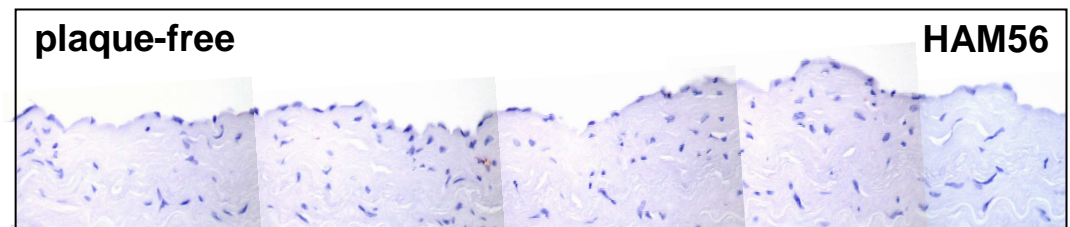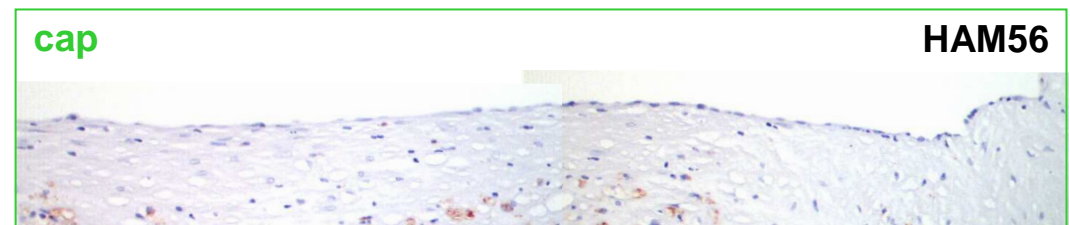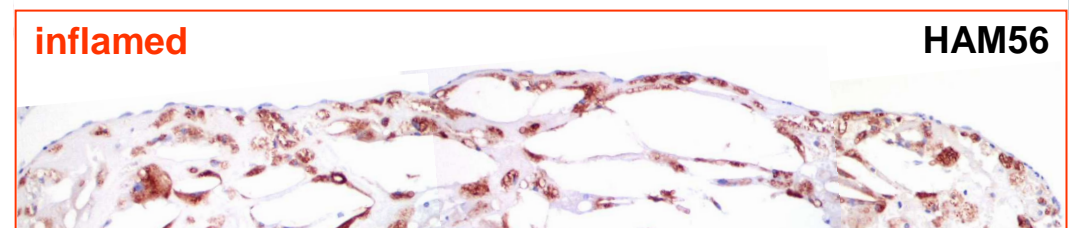

S5 Fig.

Supplement: S5 Fig — An overview, showing immunohistochemistry for macrophages (HAM56), is given for an artery with focal lesions of the advanced stage (common iliac artery, fibro-calcific plaque with signs of rupture). Rectangles within the HAM56 overview indicate the position of areas that are shown as magnification of serial sections stained for macrophages (HAM56). (PDF) [file pone.0145777.s005.pdf]
